# A new modeling environment for integrated dairy system management

**DOI:** 10.1093/af/vfz004

**Published:** 2019-03-30

**Authors:** Ermias Kebreab, Kristan F Reed, Victor E Cabrera, Peter A Vadas, Greg Thoma, Juan M Tricarico

**Affiliations:** Department of Animal Science, University of California–Davis, Davis, CA; Department of Animal Science, Cornell University, Ithaca, NY; Department of Dairy Science, University of Wisconsin–Madison, Madison, WI; U.S. Dairy Forage Research Center, Madison, WI; Ralph E. Martin Department of Chemical Engineering, University of Arkansas, Fayetteville, AR; Dairy Management Inc., Rosemont, IL

**Keywords:** dairy, simulation, systems modeling, whole farm

**Implications**

- A system approach is needed to enhance understanding of the nature of interactions among the different elements of the food and agricultural system that can be leveraged to increase overall farms’ system efficiency, resilience, and sustainability.
- The application and integration of data sciences, software tools, and systems models will enable advanced analytics for managing the food and agricultural system.
- The goal of whole-farm system modeling is to help develop sustainable dairy production systems, including the wider societal benefit of more efficient production systems while reducing negative environmental impacts.

## Introduction

Mathematical modeling techniques have been applied to study many aspects of ruminant and nonruminant animal production systems at different levels of organization. France and Kebreab (2008) argued that biology, including animal nutrition, is notable for its many organizational levels. The system can be organized from tissue level going down to cell, organelle, and molecule and could also go up to organ, organism, and herd levels—eventually simulating an entire farm or even an entire region. Although several models that describe part of the dairy system exist (e.g., Gregorini et al., 2015), whole-farm models are required to evaluate connections between system components that field research cannot practically investigate. Dairy farms are complex systems that require sophisticated management to meet sustainable production and environmental goals. Whole-farm models are valuable because they can evaluate many farm types and management practices and provide information cheaper and faster than physical experimentation. Our objective in this article is to describe a new whole-farm modeling framework that can be used to holistically assess dairy farm management practices and response to stresses (e.g., climate, disease, and resource availability) under diverse management and physical settings. The modeling system will be able to inform farm-level decisions to support more economically and environmentally sustainable dairy production systems. The animal module will be described in detail in this article, but due to space limitations, only short overviews of the other modules within the whole-farm dairy systems model framework will be presented.

## Model Categories—Strengths and Limitations

Three categories of models can potentially be applied in agricultural sciences, which are teleonomic, empirical, and mechanistic (France and Kebreab, 2008). Teleonomic modeling, which models goal-directed behavior of a system, has not yet been applied to problems in animal nutrition and physiology, though it has been used in plant and crop modeling (Thornley and France, 2007). Empirical models have been used in the field of animal nutrition for over a hundred years, particularly in development of feeding systems. These types of models use experimental data to directly quantify relationships that are usually based at a single level. For example, Niu et al. (2018) developed a series of empirical models to describe the relationship between enteric methane emissions with explanatory variables including daily feed intake, nutrient composition of the diet, and level of milk production. Empirical modeling describes data by accounting for inherent variation in the data; therefore, it is based on observation and experiment and not necessarily on understanding of underlying biological processes. Advances in precision agriculture technologies are bringing the methods and potential benefits of big data to the dairy world (Bewley, 2017). At their core, big data methods to extract information from large, often noisy datasets are empirical models. These methods can be powerful tools to support and improve animal husbandry (e.g., Stangaferro et al., 2016; Borchers et al., 2017) However, empirical models have a number of basic limitations that can only be accommodated through development and utilization of knowledge of underlying physiological and metabolic processes (Baldwin, 1995).

A mechanistic (process-based) model is constructed by looking at the underlying structure of the system under investigation, dividing it into its key components, and analyzing the behavior of the whole system in terms of its individual components and their interactions with one another (France and Kebreab, 2008). For example, Dijkstra (1994) developed a dynamic mechanistic model that simulates rumen digestion, absorption, and outflow of nutrients. The model is driven by inputs of nutrients and consists of 19 state variables representing nitrogen fractions, carbohydrates, fatty acids, and microbial biomass. Mills et al. (2001) estimated enteric methane emissions by describing the fate of excess hydrogen produced during fermentation in the rumen and hindgut, from the synthesis of lipogenic volatile fatty acids, and during microbial growth from amino acids. Because the Dijkstra (1994) and Mills et al. (2001) models were developed on biological principles, Kebreab et al. (2004) were able to integrate them and extended the integrated model to build a decision support system to analyze nutrient partitioning between the animal and its environment. If properly constructed, mechanistic models would probably give more accurate estimates of the system functions and can be extrapolated beyond the range of data used for construction. For example, Kebreab et al. (2008) showed that mechanistic models were better than empirical models in predicting methane emissions from U.S. cattle and were more suited to assessing the effectiveness of mitigation options implemented at a whole farm or national level. Although mechanistic models of ruminant digestion and metabolism have advanced our understanding of the processes underlying ruminant animal physiology, they have traditionally ignored factors such as genetic, behavior, environment/management health, and other inherent variation within and among individual animals and thus cannot assess how sources of error influence model outputs (Reed et al., 2016a). The authors further argue that predictions using Bayesian calibration of mathematical models that are expressed as probability distributions convey significantly more information than point estimates regarding uncertainty. This is still a new area of modeling in animal nutrition and needs to be further developed.

## Whole-Farm Dairy Systems Model

Existing whole-farm models, such as the Integrated Farm Systems Model (Rotz et al., 2013), DairyMod (Johnson et al., 2008), DyNoFlo (Cabrera et al., 2006), and SIMS(DAIRY) (Del Prado et al., 2011), have structural and functional limitations in representing modern farms and may not be able to take advantage of the vast amount of data currently collected on commercial farms. Existing whole-farm models are inflexible in their structure and options. For instance, researchers are unable to integrate new modules due to intractable code bases so users cannot accommodate all evolving management options. In addition, documentation and reported evaluations for existing whole-farm models are often incomplete and/or insufficient to support appropriate, intelligent use beyond the original modeling group. Moreover, existing models are restricted to relatively narrow geographic locations (Schils et al., 2007). As a consequence, these become rapidly outdated as they cannot keep pace with new technological developments such as precision agriculture or big data science. New algorithms and modern code and structure are required to advance to the next level in dairy model development and prediction goals. There is a need to develop a next-generation, whole-farm dairy model that represents a significant advancement over existing models. This will allow for holistic assessment of management practices under diverse geographic and management scenarios that may not be investigated under field conditions fully. Therefore, a model system that simulates flows of carbon, nitrogen, phosphorus, and water through the dairy system to identify ways to improve whole-farm production efficiency and minimize environmental impacts is required. In response to this situation, a Ruminant Farm System modeling environment should be developed by employing modern computer coding practices, emphasizing model clarity and adaptability to achieve flexibility and practical applications in research and industry. Furthermore, this next-generation model should be able to incorporate sensor data and take full advantage of big data artificial intelligence, which are part of modern dairy farm systems. Data mining and deep learning should guide model development and validate performance. To integrate information gained from on-farm data streams into a system analysis, a process-based simulation model core that starts with a mass and energy balance of farm nutrient, energy, and water cycles is essential.

**Figure 1.**
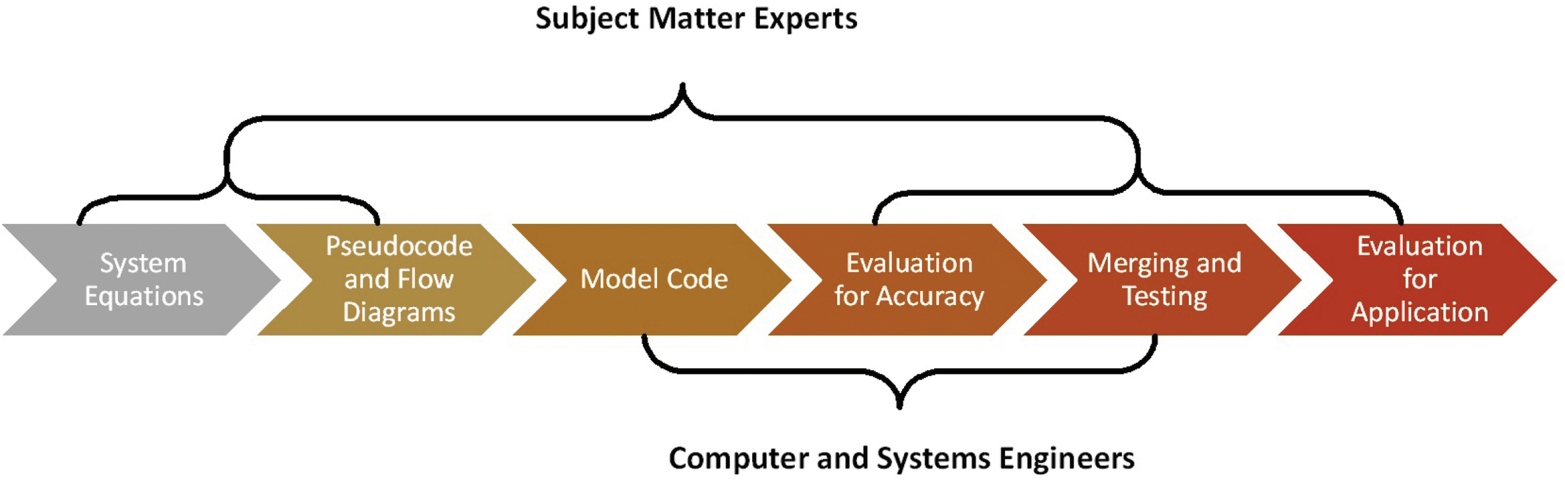
Diagram illustrating the interaction between subject-matter experts and programmers in the model development process.

Rigorous adherence to well-defined, modern model development methodologies is essential for a successful next-generation dairy systems model that avoids the pitfalls of its predecessors. Well-structured and well-documented code is fundamental to model transparency; intelligent, widespread application; and adaptation to future knowledge. Jones et al. (2001) identified seven facets of a modular structure that will lead to model adaptability, interdisciplinary use, and endurance. Examples of effective implementation of modular model development exist in other disciplines (Jones et al., 2003; Arnold et al., 2012) but have not yet seen success in animal agriculture systems. For this reason, we have defined the following systematic, interdisciplinary model development and documentation methodology (Figure 1):

• *Description of the underlying biological system*: Subject-matter experts in disciplines relevant to each module draw information from existing models and recent literature to describe all necessary processes with appropriate mathematical algorithms, emphasizing simplicity as a driving paradigm.
• *Development of pseudocode and information flow charts*: Detailed, uniformly formatted documentation for all parts of the model serve as the documentation foundation. Description of and references for each simulated process in the pseudocode combined with multilevel information flow diagrams will facilitate model clarity and use.
• *Translation of pseudocode into code*: Pseudocode and flow charts will be translated into code by trained programmers in collaboration with discipline experts. Emphasis should be placed on the clarity of code through code structure and logical and detailed commenting.
• *Evaluation of submodel functions with existing datasets*: As development proceeds, module evaluation for individual and combinations of processes using existing experimental and literature-based datasets is essential. Evaluation datasets should stress the system by pushing the model to operate at the edges of the biological limits at which the underlying process models were developed. All model evaluation datasets will be made available to the public.
• *Incorporation of submodules into modules and systems model*: When the basic function of submodules and modules have been verified, routine incorporation into the larger systems model and retesting after merging should occur.
• *Evaluation of model structure and outputs for user application*: As submodules and modules progress, simulation of partial and complete model function should be demonstrated and further evaluated with new datasets.

The model development strategy is designed to produce clear documentation and a readable, readily adaptable codebase of a modular dairy farm system model.

As described by Jones et al. (2001), there are many benefits of the modular format which, we believe, will help prevent limitations of past whole-farm models. The same paradigm applied to each module and submodule strengthens model clarity and adaptability by enabling individual modules and submodules to be evaluated, updated, or exchanged for new ones as knowledge of a particular farm component advances. Furthermore, modularity ensures future updates to specific parts of the dairy systems model can be made without significantly disrupting other modules and for simulated processes to be replaced by knowledge of constituent farm, where available. The latter attribute will be increasingly valuable as a means to integrate the growing streams of sensor data collected on farms for improved whole-farm decision-making.

Another key advantage of the adaptable, modular nature of the model is that the level of detail required for and from model inputs and outputs can be tailored to meet the needs of the investigator; this feature will contribute to broader adoption and widespread application. For example, someone interested in assessing the efficiency of a specific crop rotation may not be interested in specifying reproductive protocols for each animal or group of animals or in simulated results of reproductive efficiencies. In this case, standard sets of inputs and outputs governing the animal management portion of the model can be selected by the user with more specialized user input directed toward the Crop and Soil module.

By combining a range of scientific expertise with experienced programmers, the resulting model will have a sound foundation in both modern science and program structure that can be widely used and easily adapted as our knowledge of the dairy system advances.

**Figure 2.**
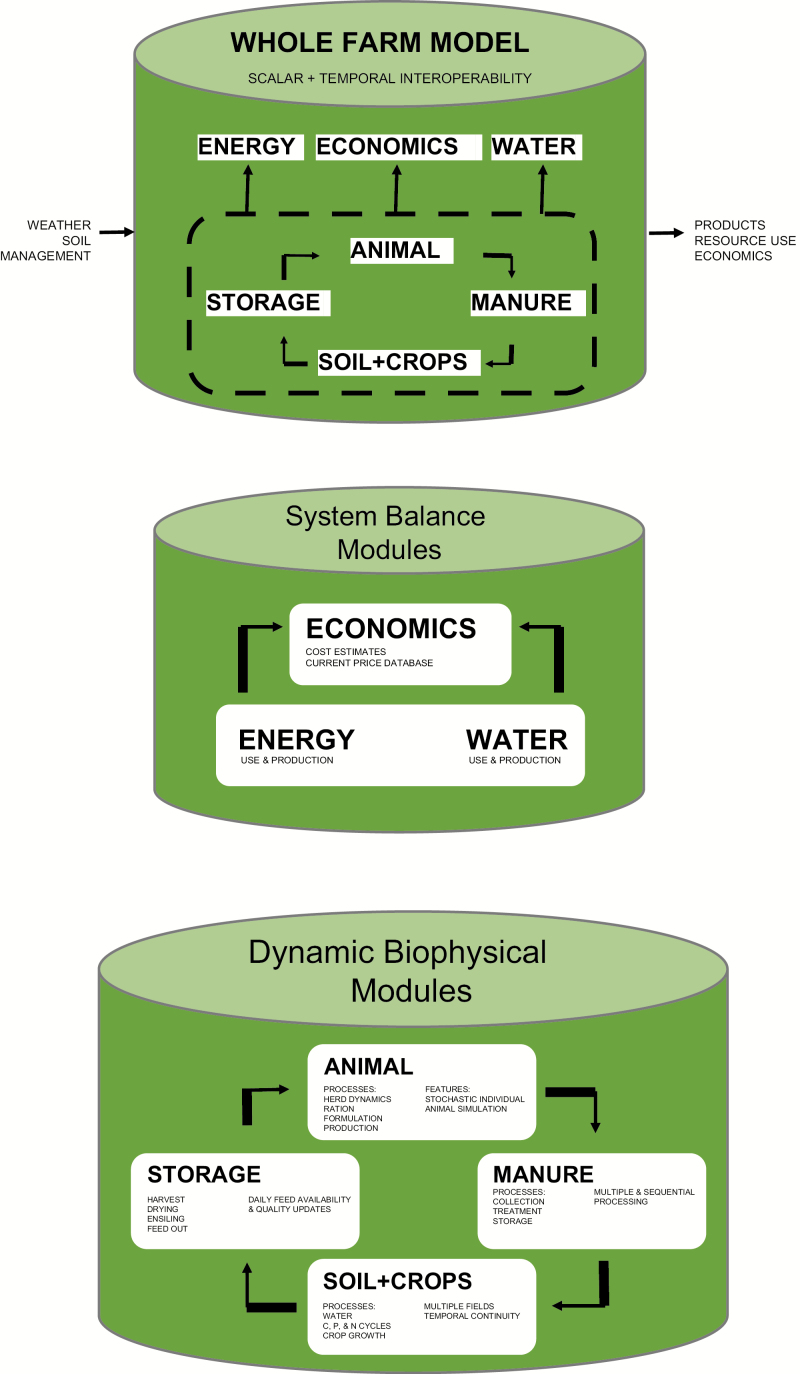
Model schematic representing a whole-farm system including the four integrated biophysical modules of Animal, Manure, Soil and Crop, and Storage and the three system balance modules of Water, Energy, and Economics.

The model foundation will be four integrated biophysical modules (Animal, Manure, Soil and Crop, and Feed Storage) that follow carbon and nutrients (nitrogen, phosphorus, potassium) as they cycle through the dairy system and three modules (Economic Accounting, Energy, and Water Balance) that synthesize information from each of the four biophysical modules (Figure 2).

The biophysical modules will simulate inputs, transactions, exports, and losses of water, carbon, nitrogen and phosphorus with process-based modeling to account for social (management), built (infrastructure), and environmental (weather) impacts on biological (crop and animal growth; milk production) and geophysical (soil emission) processes that occur on the dairy farm. Major inputs to the four biophysical modules include daily weather (precipitation, air temperatures, wind speed, humidity, radiation); soil physical and chemical properties; numbers and breeds of cattle; and management practices governing manure management, crop production and storage, and animal breeding, handling, and milking. The Economic, Energy, and Water modules use inputs of management practices and outputs from the biophysical modules alongside adjustable libraries of monetary and energetic costs of common practices and commodities to enable integrated evaluation of dairy management systems for environmental, social, and economic sustainability. Simulating the entire dairy nutrient, water, and energy cycle with process models will enable whole-system impact assessment of management and environmental changes. Examples of scenario assessments of which the model will be capable include:

1. How does herd health management (e.g., disease incidence, nutrition) affect animal productivity and nutrient use efficiency, nutrient excretion in manure, potential nutrient recovery in manure processing, and nutrient fate in crop uptake and loss after application of manure?
2. How do changes in soil health parameters (erosion, soil carbon) influence crop growth and farm productivity? What are the impacts of environment, soils, and management on crop feed quality?
3. How do farm strategies (e.g., large confinement operations vs. small grazing farms) affect whole-farm sustainability metrics including food production, energy use or production, and water and nutrient use efficiency and availability?

In addition, the model will be able to assess these management and geophysical scenarios under varying climate conditions, including future climate change projections.

### Animal Module

**Figure 3.**
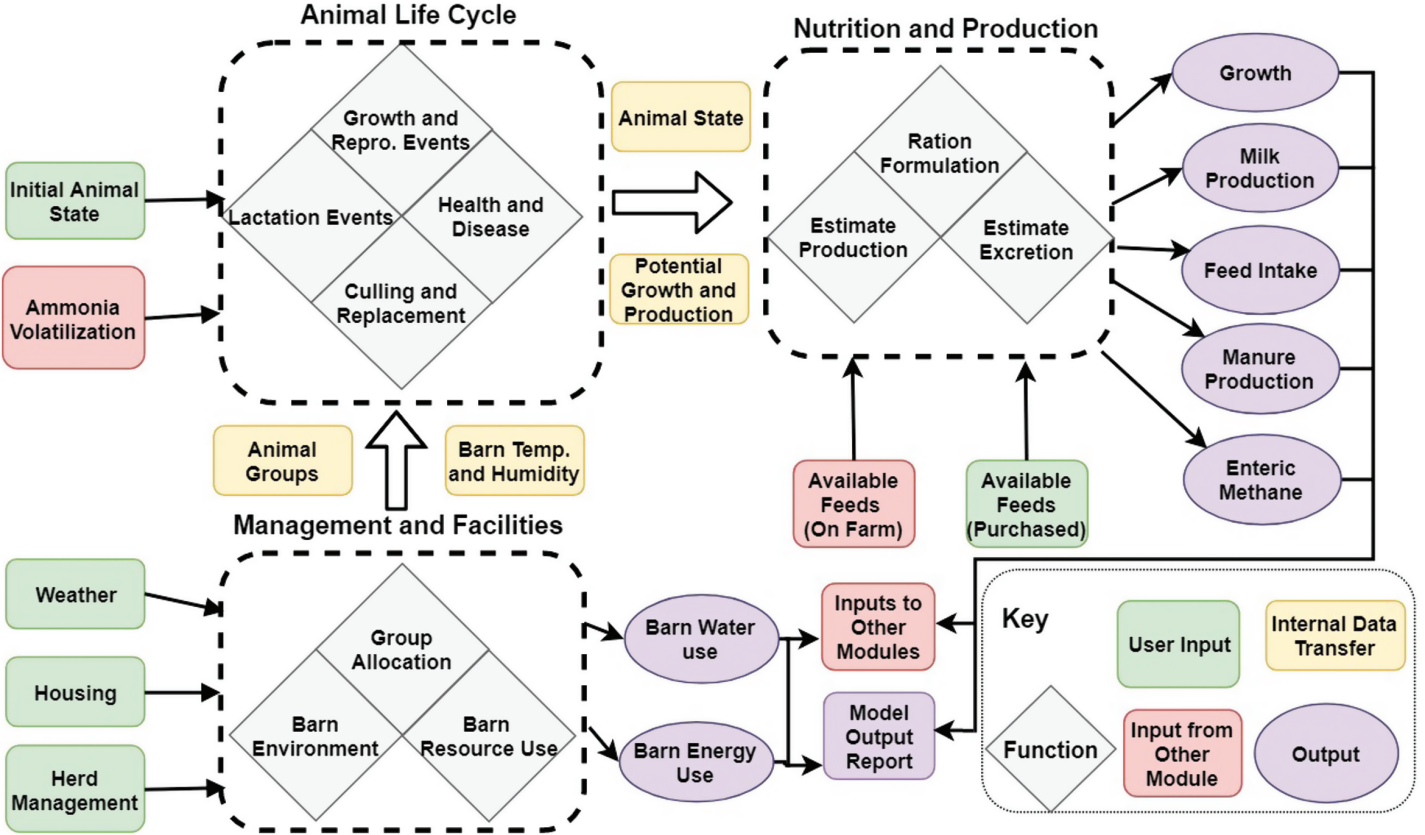
Schematic of information flows in the Animal module.

This module will consist of three primary submodules: 1) Animal Life Cycle, 2) Nutrition and Production, and 3) Management and Facilities (Figure 3).

The Animal Life Cycle submodule will be a stochastic Monte-Carlo model of a dairy cow’s life events from birth to culling or death. The animal module takes information about feed, weather, and management from the user and other modules to stochastically simulate growth, production, and reproduction of individual cows daily. The life cycle submodule simulates animal states and herd dynamics, which can then be used for ration formulation, milk and manure production, and economics. Future performance is uncertain and dependent on probabilistic outcomes, which is better captured by stochastic probabilistic models (Calsamiglia et al., 2018) when compared with deterministic or conventional models. Stochastic models simulate probabilistic distribution of events and also reports a distribution of possible outcomes, which better represent reality. Prior distributions inform posterior outcomes and include all the possible interactions among parameters (Baudracco et al., 2013). Different than dynamic programming (De Vries, 2006) or Markov-chain-based (Cabrera, 2012) models, which have been widely used to simulate dairy herds, stochastic Monte-Carlo models have the advantage of simulating each animal individually and still accommodate the interactions of the herd dynamics. With improved computational power, big continuous data for parameterization, and opportunity for individual- and herd-level permanent operational and strategic decision-making, stochastic Monte-Carlo models seem to be the most adequate framework (Kalantari et al., 2016; Calsamiglia et al., 2018). A simulation example is as follows: a calf is generated with a selected breed (e.g., Holstein), semen type (e.g., sexed semen), and birth weight (e.g., 40.8 kg). Sex is randomly determined with probabilities of the chosen semen type (e.g., 90% female for sexed semen). Male calves are sold; female calves enter the herd. The calf’s weight changes based on the breed’s average daily gain (e.g., 0.9 kg/d). When the calf reaches 400 d, first ovulation occurs with a lognormal distribution of logN (19, 11) and starts an estrus cycle with length distributed as N (21, 4). An example breeding method is the estrus detection-artificial insemination method in which the probability of heat detection during estrus is set at 60%. When heat is detected, insemination proceeds with a conception rate of 33.9% for the first insemination and decreases by 2.6% for each subsequent insemination. After insemination, there are three pregnancy diagnoses on days 32, 91, and 200. Between the first and second, and second and third diagnoses, the chances of daily pregnancy loss are 0.96% and 0.17%, respectively. Gestation length is distributed as N (278, 6). A cow not detected as in heat or pregnant goes into the next estrus cycle. If the heifer grows to 650 d or the cow reaches over 300 d in milk without getting pregnant, it is culled. Lactation starts at calving and follows either Wood’s or MilkBot curve with specific breed, parity, and production parameters (Li et al., 2018). Six additional culling reasons are implemented: lameness, injury, mastitis, other diseases, udder problems, or unknown. For each reason, the probability of culling is compared with a stochastic number from U (0, 1) to determine whether the cow is culled in its lifetime. The age of culling is determined by the inverse comparison of another random draw from U (0, 1) with an empirical cumulative distribution function for the probability of culling occurring on each day (Kalantari et al., 2016). Genetics will also be included as factors that influence the animal’s life cycle, and the model will have the ability to utilize parameters set by the user or import herd-specific values.

The Nutrition and Production submodule will be further subdivided into two primary user-driven options: 1) ration formulation to meet potential milk production and 2) user-defined ration to mechanistically predict milk production (nutrient-based prediction system). The former feeding strategy uses linear programming to find the optimal diet that meets the nutrient requirements for a given milk production. It includes constraints regarding available feeds, as well as animal, feed, and specific nutrient intake constraints. Additional nonlinear ration optimization strategies including maximizing income-over-feed-costs and minimizing nitrogen excretion will also be investigated to diversify feed-strategy options. Nutrient requirements are based on those of the upcoming release of the eighth edition of Nutrient Requirements of Dairy Cattle (National Academy of Science, Medicine and Engineering, in preparation) and the most recent version of the Cornell Net Carbohydrate and Protein System (http://blogs.cornell.edu/cncps/home/). The second option in the Nutrition and Production submodule uses a mechanistic approach. Several mechanistic models are available (e.g., Kebreab et al. 2004; Gregorini et al., 2015) that can be integrated for use in the submodule. However, models that are capable of representing technologies such as rumen protected amino acids to improve protein utilization in dairy cattle (e.g., Reed et al., 2016b) or impact of feed additives in reducing methane emissions are preferred.

The individual animal structure of the Animal Life Cycle submodule will give the Nutrition and Production submodule flexibility to investigate feeding strategies that range from daily formulation of individual cow rations to group feeding with ration updates at any time scale. By further giving the user an opportunity to define their own ration, the second feeding strategy allows for investigations into system-wide impacts of specific diets. The model will allow estimates of how much water is likely to be consumed for a given level of production using databases on water requirements for crops that are included in the ration. The model will also estimate the amount of manure excreted based on the work by Appuhamy et al. (2018). Furthermore, the trade-off of methane emitted through enteric fermentation or shift to manure based on the amount of volatile solids excreted can also be evaluated as it will have an impact on the amount of energy generated if the manure were to be used as input for biogas production.

The Management and Facility submodule is a barn-level simulation that focuses on the interaction of environment and human intervention with the herd. It processes the management choices together with environmental factors to inform the Animal Life Cycle iteration and output energy and water use to the system balance modules. The Management and Facility submodule will use weather information, on-farm facility information like barn structure, bedding, cooling system, and parlor type, and management practices like grouping strategy and replacement management to determine inputs to the Animal Life Cycle and Nutrition and Production submodules. For example, it will process the weather information and barn ventilation/insulation properties to determine the potential for cold/ heat stress to affect dry matter intake, water consumption, and production. In the absence of direct user input of pen for each animal, this submodule will also include algorithms for sorting cows into groups based on different nutritional grouping strategies.

### Soil and Crop Module

The Soil and Crop module represents a two-dimensional soil profile and allows the user to specify the number and depths of soil layers. It will use climate inputs to simulate soil temperature and hydrology, including evapotranspiration, surface runoff (in pseudo 3D with areal extent and layers down), leachate, and soil moisture. It will also simulate soil carbon, nitrogen, phosphorus, and potassium dynamics with different chemical pools to represent short and long-term dynamics and fate, including loss in water and air emissions (carbon dioxide, ammonium, nitrous oxide). The crop equations will represent the major crops grown on dairy farms, including corn, alfalfa, grass (pasture), legumes (clover), small grains, and soybeans. Equations will simulate crop growth and development (with appropriate limitations due to water, temperature, and nutrient availability stresses), nutrient uptake and removal, and residue decomposition. Equations will also simulate feed quality aspects of crops as needed for the animal module. Existing, well-established equations for the soil and crop module will be used, so simulations are consistent with other widely used soil and crop production models (e.g., SWAT, DayCent). One particular model aspect that will draw from existing models is pasture production and animal grazing. Models in the UK, Australia, and New Zealand (e.g., SimsDairy) are well developed to simulate the intricacies of pasture growth and animal feeding routines. These will be adapted for use in the new model framework. The model will be able to simulate a number of soil type and crop combinations, so the biophysical variability inherent to a dairy farm fields and crop rotations is well represented. Finally, the module will include an irrigation component to evaluate new irrigation strategies to maximize water use efficiency.

### Feed Storage Module

The Feed Storage module will simulate carbon and nitrogen dynamics during feed harvest and storage. The major component of this module will estimate nutrient loss during ensiling as a function of crop type, silo type, packing parameters, climate, moisture, inoculant, feed removal from storage, and feedout conditions. Carbon and nitrogen loss pathways include greenhouse gas and volatile organic carbon emissions, leachate, and degradation of feed quality that can be passed into other modules. In particular, loss to spoilage and protein degradation will be of critical importance to feed quality and pathogen load moving into the animal module. In addition to simulating changes to feed composition during storage, this module will keep track of feed stock and storage space availability for communication with the Crop and Soil and Animal modules.

### Water Balance Module

The Water Balance module is an overarching module that will use information from the four biophysical modules as well as user input on farm practices to simulate water use and generation by cattle, water use, and capture in cattle management (e.g., milking parlor wash water), water captured and used in manure management, water losses and additions during manure handling and storage as well as stormwater management, and water use in cropping systems, including new and improved irrigation technologies to improve water use efficiency.

### System Integration

All the modules will be linked and organically integrated to ensure consistency and transparency and to streamline all the data input and output flows. Although we consider a single-farm model framework, externalities, such as climate and weather conditions, government policy and incentive, connected water systems (e.g., precipitation, evapotranspiration, runoff, groundwater, etc.), energy systems (e.g., power grids that could accept renewable electricity generated from dairy manure), and food systems (e.g., agricultural operations, including conservation practices, of related farms) will be systematically integrated and accounted for throughout the “cradle-to-grave” life cycle. Critical components of the systems at the local, regional, and global scales, as well as the interscale synergies and effects, should be captured in the proposed modeling framework.

## Summary

Our next-generation dairy systems model will fill a major gap in available tools to advance the frontier of knowledge in developing dairy systems for sustainable food production, environmental quality, water and nutrient use efficiency, and energy efficiency and production. It will provide novel information to the scientific community about managing dairy production at a farm system level instead of optimizing single-farm operations. The model will also help scientists better inform farmers, practitioners, and industry and policy leaders on the environmental and economic impacts of adding, removing, or changing one or multiple dairy farm practices.

## About the Authors

**Dr. Ermias Kebreab** is Associate Dean and holds the Sesnon Endowed Chair in Sustainable Animal Agriculture at the University of California–Davis. Dr. Kebreab conducts research in nutrition modeling of biological systems and conducts experiments to reduce the impact of animal agriculture on the environment, including the sustainable use of feed additives. He is a contributing author to 2019 IPCC update, and chairs the feed additive committee of the Food and Agriculture Organization. Dr. Kebreab received the AFIA Ruminant Nutrition Award in 2014. Dr. Kebreab earned a BSc from the University of Asmara, Eritrea, and MSc and PhD from the University of Reading, UK.

**Dr. Kristan F. Reed** is an Assistant Professor of Dairy Cattle Nutrition in the Animal Science Department at Cornell University. Her research aims to improve dairy production efficiency and sustainability through development and use of models that simulate the behavior of dairy farming systems under diverse biological, environmental, and management conditions. Dr. Reed received her BS in Animal Science from Cornell University and PhD in Animal Biology from the University of California at Davis.

**Dr. Victor E. Cabrera** combines applied research, interdisciplinary approaches, and participatory methods to deliver practical, user-friendly, and scholarly decision support tools for dairy farm management. These scientific tools are improving dairy farm profitability, environmental stewardship, and long-term sustainability of the dairy farm industry.

**Peter Vadas** is a research scientist with USDA-Agricultural Research Service at the Dairy Forage Research Center in Madison, WI. His research focuses on nutrient management in agricultural systems, especially developing decision tools and models for nutrient fate and transport in animal production. He has conducted lab and field research to generate the data needed to improve existing field-, farm-, and watershed-scale models that are used to help manage agricultural nitrogen and phosphorus. He is originally from Maryland and obtained his BS in Environmental Science from Virginia Tech and MS and PhD in Soil Science from the University of Delaware.

**Greg Thoma**, PhD, is the Bates Teaching Professor of Chemical Engineering at the University of Arkansas. He led numerous food and agriculture life cycle assessment projects: milk, cheese, milk delivery systems, yoghurt, swine, poultry, corn, and beef. He developed process models coupled with life cycle assessment models for swine and poultry. He serves on the steering committee for the Swiss National Research Program, “Healthy Nutrition and Sustainable Food Production.” He serves as subject editor for Agriculture for the International Journal of Lifecycle Assessment. He has been active with the Livestock Environmental Assessment and Performance (LEAP) Partnership since its inception.

**Dr. Juan M. Tricarico** is Vice-President for Sustainability Research at Dairy Management Inc. where he supervises the Innovation Center for U.S. Dairy sustainability research team to drive trust and sales in dairy through improved sustainability science. Dr. Tricarico holds a licentiate degree from Universidad C.A.E.C.E. in Buenos Aires, Argentina, and a PhD from the University of Kentucky. He worked at Alltech Inc. where he developed and evaluated feed additives focused on ruminal fermentation and its implications on ruminal microbiology, carbohydrate fermentation, and nitrogen utilization in ruminants. Dr. Tricarico is President-Elect 2018–2019 for the Council of Agricultural Science and Technology (CAST).
